# P-659. Pilot Phase Results of the ARIA Study: Acute Respiratory Illness among Adults 50 and Older in the Outpatient Setting

**DOI:** 10.1093/ofid/ofaf695.872

**Published:** 2026-01-11

**Authors:** Jennifer L Kuntz, Holly C Groom, Jennifer K Meece, John F Dickerson, Richard A Mularski, Weiming Hu, Maureen O’Keeffe-Rosetti, Nicola P Klein, Karen Jacobson, Amy Wiesner, Courtney Oxandale, Lisa Glasser, Sudhir Venkatesan, Carla Talarico, Mark A Schmidt

**Affiliations:** Kaiser Permanente Center for Health Research, Portland, Oregon; Kaiser Permanente Center for Health Research, Portland, Oregon; Marshfield Clinic Research Institute, Marshfield, Wisconsin; Kaiser Permanente Center for Health Research, Portland, Oregon; 1. Kaiser Permanente Center for Health Research, Portland, Oregon, Portland, Oregon; Kaiser Center for Health Research, Portlannd, Oregon; Kaiser Permanente Center for Health Research, Portland, Oregon; Division of Research Kaiser Permanente Vaccine Study Center, Oakland, California; Kaiser Permanente Vaccine Study Center, Division of Research, Oakland, California; Kaiser Permanente Vaccine Study Center, Division of Research, Oakland, California; Kaiser Permanente Vaccine Study Center, Division of Research, Oakland, California; AstraZeneca, Wilmington, DE; Medical and Payer Evidence Statistics, BioPharmaceutical Medical, AstraZeneca, Cambridge, UK, Cambridge, England, United Kingdom; Vaccines and Immune Therapies, BioPharmaceuticals Medical, AstraZeneca, Gaithersburg, MD, USA, Gaithersburg, Maryland; Center for Health Research, Kaiser Permanente Northwest, Portland, Oregon

## Abstract

**Background:**

The Acute Respiratory Illness in Adults (ARIA) Study is a two-year prospective study, with a pilot phase intended to determine the feasibility of assessing the burden of acute respiratory illness (ARI) in adults. In this analysis, we describe the pilot phase results from ARIA among older adults treated in the outpatient setting between November 18, 2024 and March 31, 2025.

**Figure:**
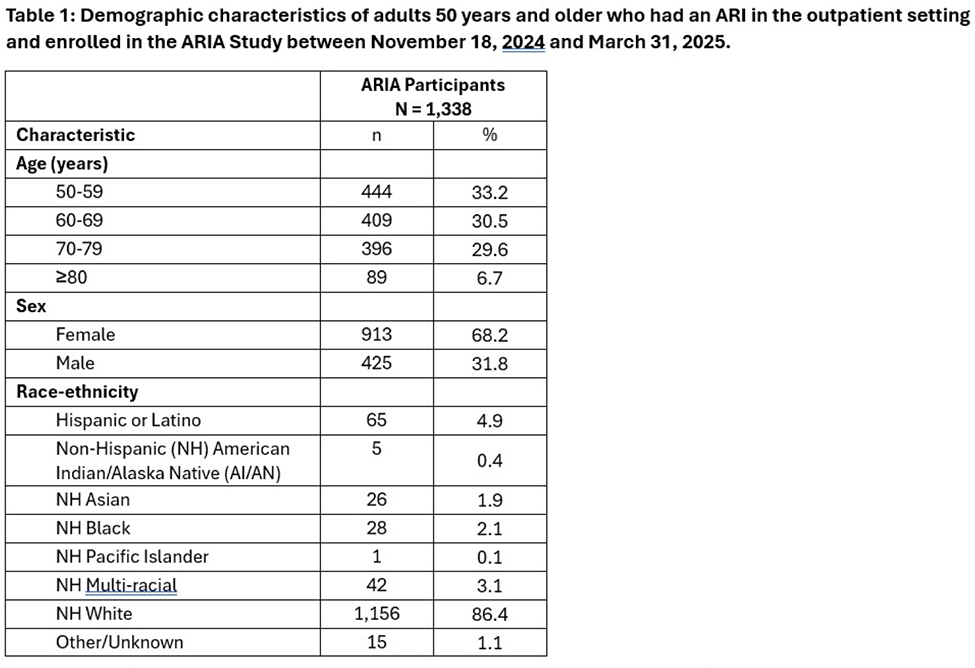

**Figure:**
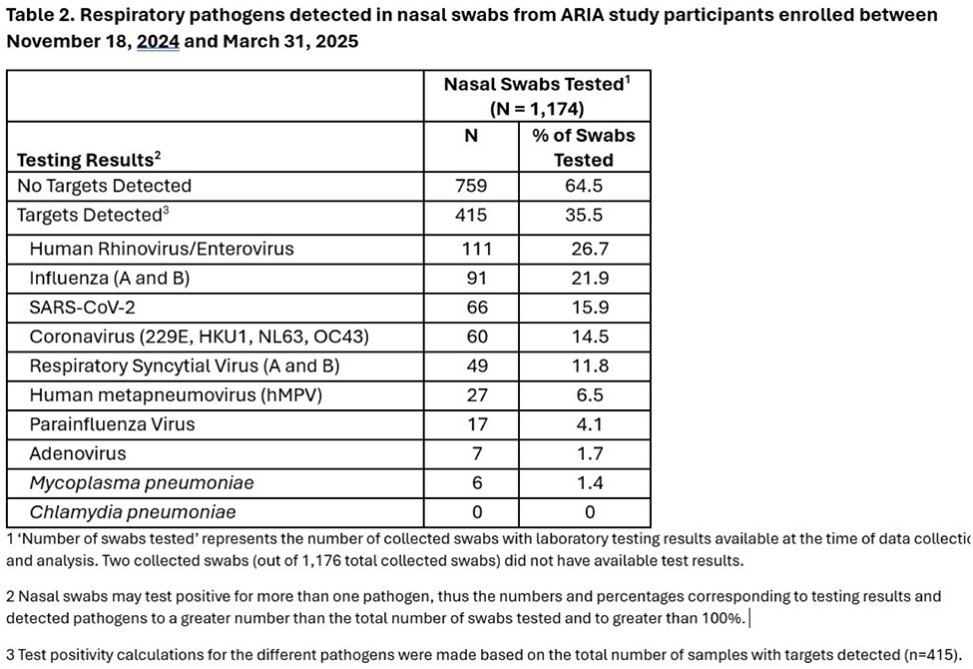

**Methods:**

We recruited Kaiser Permanente Northwest members 50 years and older who had an outpatient or telehealth visit for ARI between 11/18/2024 and 3/31/2025. Enrolled participants self-collected nasal swabs, which we tested through real-time PCR using the GenMark ePlex RP2 multiplex panel at Marshfield Clinic Research Institute. We describe population characteristics of participants and nasal swab testing results.

**Results:**

Among 16,830 eligible individuals, we enrolled 1,338 (8.0%) participants, of whom 1,176 (88%) returned nasal swabs. Population characteristics are shown in Table 1. Among tested swabs, pathogens were detected in 415 (35.5%), with the most common being rhinovirus/enterovirus (n=111; 26.7%), influenza (n=91; 21.9%), SARS-CoV-2 (n=66; 15.9%), and other coronaviruses (14.5%). Respiratory syncytial virus (RSV A or B) and human metapneumovirus (hMPV) were detected in 11.8% (n=49) and 6.5% (n=27) of swabs, respectively (Table 2). Influenza test positivity rate peaked during two time periods (12/15/25 to 1/04/25 and 1/19/25 to 3/01/25); RSV peaked from 2/16/25 to 3/01/25; and hMPV test positivity rate increased towards the end of our observation window (3/16/25 to 3/29/25).

**Conclusion:**

This pilot phase analysis of the ARIA study demonstrates the feasibility of assessing pathogen positivity among enrolled outpatients, with rates reflecting those observed in the community at large for the same time period in the 2024-2025 season. Since hMPV circulation often occurs later than influenza and RSV, ongoing data collection will improve the characterization of ARI epidemiology, especially that due to hMPV. Over the next two years, the ARIA study will provide vital real-world evidence about the incidence, severity, and impact of viral ARI among older adults in the outpatient setting to help inform the development of prophylactic and treatment options.

**Disclosures:**

Jennifer L. Kuntz, MS, PhD, Astra Zeneca: Grant/Research Support|Moderna, Inc.: Grant/Research Support|Pfizer: Grant/Research Support Holly C. Groom, MPH, AstraZeneca: Grant/Research Support|Moderna: Grant/Research Support Jennifer K. Meece, PhD, CSL Seqirus: Grant/Research Support|GSK: Grant/Research Support|ModernaTX: Grant/Research Support John F. Dickerson, PhD, AstraZeneca: Grant/Research Support|HilleVax: Grant/Research Support|Moderna: Grant/Research Support Richard A. Mularski, MD, MSHS, MCR, AstraZenica: Grant/Research Support|Pfizer, Inc: Grant/Research Support|Sanofi: Grant/Research Support Weiming Hu, MS, AstraZeneca: Grant/Research Support|Janssen: Grant/Research Support Maureen O'Keeffe-Rosetti, MS, Astra Zeneca: Grant/Research Support|HilleVax: Grant/Research Support|Moderna: Grant/Research Support|Pfizer: Grant/Research Support Nicola P. Klein, MD, PhD, AstraZeneca: Grant/Research Support|Centers for Disease Control and Prevention: Grant/Research Support|GlaxoSmithKline: Grant/Research Support|Janssen: Grant/Research Support|Merck: Grant/Research Support|Moderna: Grant/Research Support|Pfizer: Grant/Research Support|Sanofi Pasteur: Grant/Research Support|Seqirus: Grant/Research Support Karen Jacobson, MD, MPH, AstraZeneca: Grant/Research Support Lisa Glasser, MD, AstraZeneca: Stocks/Bonds (Public Company) Sudhir Venkatesan, MPH, PhD, AstraZeneca: Stocks/Bonds (Public Company) Carla Talarico, PhD, MPH, AstraZeneca: Stocks/Bonds (Private Company) Mark A. Schmidt, PhD, MPH, AstraZeneca: Grant/Research Support|HilleVax: Grant/Research Support|Janssen: Grant/Research Support|Moderna: Grant/Research Support

